# *Mycoplasma genitalium* infection and risk of ovarian cancer: a systematic review and meta-analysis

**DOI:** 10.1590/1806-9282.20250562

**Published:** 2025-09-19

**Authors:** Hui Li, Liyuan Shi, Chuan Wang, Li Tian, Yufan Yuan, Ling Jia, Sha Wang

**Affiliations:** 1 Shijiazhuang Fourth Hospital, Department of Gynecology – Shijiazhuang, China.; 2 Shijiazhuang Traditional Chinese Medicine Hospital, Department of Gynecology – Shijiazhuang, China.; 3 Shijiazhuang Traditional Chinese Medicine Hospital, Operating Room – Shijiazhuang, China.; 4 Shijiazhuang Fourth Hospital, Department of Nursing – Shijiazhuang, China.

## INTRODUCTION

Ovarian cancer is one of the most lethal gynecological malignancies worldwide. In 2022, an estimated 324,398 new cases and 206,839 deaths were reported, making it the eighth most common cancer among women and the leading cause of gynecological cancer mortality^1^. The high fatality rate is due to its asymptomatic early stages and lack of effective screening, leading to late diagnoses^2^. Established risk factors include genetic predisposition (BRCA1/2 mutations), family history, and reproductive factors such as nulliparity and early menarche^3^. Recently, sexually transmitted infections (STIs) have emerged as potential contributors to ovarian cancer risk, warranting further investigation^3^.

STIs are increasingly recognized as oncogenic factors in reproductive cancers^4^. While human papillomavirus (HPV) is well known for its role in cervical cancer^5^, emerging research suggests that *Chlamydia trachomatis* and *Mycoplasma genitalium* (MG) may contribute to ovarian carcinogenesis by inducing chronic inflammation and immune dysregulation^6^. Since STIs often remain asymptomatic in the upper reproductive tract, their role in malignancy could be underestimated^7^. MG, first identified in the 1980s, is a sexually transmitted bacterium linked to non-gonococcal urethritis in men and cervicitis and pelvic inflammatory disease (PID) in women^8^. Its global prevalence ranges from 1 to 3%, with higher rates in high-risk populations^9^. Notably, *Mycoplasma genitalium* infection (MGI) is persistent and often resistant to antibiotics^10^, leading to prolonged inflammation in the upper genital tract, including the ovaries^11^. This chronic inflammation may drive carcinogenesis through cellular damage, DNA mutations, and apoptosis disruption^12^.

Despite growing research, the association between MGI and ovarian cancer remains unclear^6^. While some studies report a significant link, others find no correlation, highlighting the need for further investigation. Given these inconsistencies, this study aimed to systematically review and meta-analyze available evidence to clarify the relationship between MGI and ovarian cancer risk, providing insights for future research.

## METHODS

### Literature search strategy and study selection

A comprehensive literature search (from inception to January 30, 2025) was conducted across Scopus, MedLine/PubMed, Web of Science, and Embase to identify studies on the association between MGI and ovarian cancer risk. The search used a combination of keywords and Medical Subject Headings (MeSH) terms, including "*Mycoplasma genitalium*," "sexually transmitted infections," "bacterial infections," and "ovarian cancer," with no restrictions on language, publication date, or geography. Additional sources included gray literature (OpenGrey and ProQuest Dissertations and Theses Global), preprint repositories (medRxiv, bioRxiv, and Research Square), and manual reference lists. Eligible studies included observational designs (case–control, cohort, and cross-sectional) reporting adjusted odds ratios (ORs) or relative risks (RRs) with 95%CIs. Experimental studies, case reports, reviews, and those with insufficient data or unclear diagnostic criteria were excluded.

### Data extraction and quality assessment

Two independent reviewers (HL and LS) performed data extraction using a predefined extraction form. The extracted data included study characteristics (author, year, country, study design, and sample size), population characteristics (age, sex, and study population), exposure (diagnostic criteria for MGI), outcome (ovarian cancer diagnosis), and effect measures (adjusted ORs or RRs with 95%CIs). Any discrepancies between the reviewers were resolved through discussion or by consulting with the principal authors (LJ and SW). The quality of the included studies was assessed using the Newcastle-Ottawa Scale (NOS) for observational studies^13^. Studies were evaluated based on the selection of participants, comparability of groups, and outcome assessment. Studies scoring 7 or higher on the NOS were considered of high quality.

### Statistical analysis

The meta-analysis was conducted using the DerSimonian and Laird random-effects model (REM) to account for heterogeneity across studies^14^. The primary outcome was the pooled adjusted OR for the association between MGI and ovarian cancer. Heterogeneity was assessed using the I^2^ statistic, with values above 50% indicating substantial heterogeneity. In addition to the pooled effect size and its 95%CI, we calculated the 95% prediction interval (PI) to estimate the range within which the true effect sizes of future studies are expected to fall. For this, we used the standard formula incorporating the pooled effect, its standard error, and between-study variance (τ^2^)^15^. Sensitivity analyses were performed by excluding studies with a high risk of bias and by restricting the analysis to high-quality studies. Additionally, cumulative meta-analyses were conducted to examine the stability of the association over time. Subgroup analyses were performed based on study design, geographic region, and population characteristics. Publication bias was assessed through visual inspection of funnel plots and quantitatively using Egger's regression test and Begg's test. If significant publication bias was detected, the Duval and Tweedie's trim-and-fill method was applied to adjust the pooled estimates. All statistical analyses were conducted using Stata version 17.0 (StataCorp, College Station, TX, USA), with a significance level set at p<0.05. To report the findings of this study, we followed the Preferred Reporting Items for Systematic Reviews and Meta-Analyses (PRISMA) guidelines(Supplementary Table 1).

## RESULTS

### Literature search and characteristics of studies included

The systematic search identified 803 potential studies: 777 from various databases and 26 from additional sources. After removing duplicates, 589 unique records were screened based on titles and abstracts, resulting in 570 exclusions. A total of 19 full-text articles were further assessed for eligibility, with 11 excluded due to a lack of control groups (n=2) and non-original data (n=9). Ultimately, eight studies met the criteria for inclusion in both qualitative and quantitative analyses (Supplementary Figure 1). The included studies varied in terms of design, spanning case–control and cohort studies, and represented diverse geographic regions. The main characteristics of the included studies are demonstrated in Table 1. The included studies, conducted between 2010 and 2023, encompassed a multinational scope, including Sweden, China, Iran, Poland, Finland, and the United States. The majority of studies (n=6) used a nested case–control design; two additional studies employed a simple population-based case–control design. The detection of MGI was accomplished using diverse methodologies: serological assays (n=5), polymerase chain reaction (PCR) (n=2), and immunohistochemistry (n=1). Most studies (according to the NOS) were of high methodological quality; only two received a moderate rating. Participant ages ranged from 18 to 87 years across studies. More details are presented in Table 1.

**Table 1:** Main characteristics of the studies included.

Included studies	Implementation year	Country	Diagnostic methods	Mean ages or age range		Patients with ovarian cancer		Controls		NOS score
				Cases	Controls	Number	Mycoplasma positive	Number	Mycoplasma positive	
Idahl et al. (2010)🕈 [Table-fn TFN2]	1994–2001	Sweden	PCR	58.9	55.9	52	0	134	0	High
Yang et al. (2010)✞ [Table-fn TFN1]	1997–2005	China	Immunohistochemistry	NR	NR	109	47	30	0	Moderate
Idahl et al. (2011)🕈 [Table-fn TFN2]	1993–2001	Sweden	Serology	31–82	18–87	45	4	180	6	High
Dadashi et al. (2016)✞ [Table-fn TFN1]	2014–2015	Iran	PCR	NR	NR	62	0	62	0	Moderate
Fortner et al. (2019)🕈 [Table-fn TFN2]	2015–2016	USA	Serology	34–81	35–80	337	17	337	9	High
Idahl et al. (2020)🕈 [Table-fn TFN2]	1992–2000	Sweden	Serology	30–81	30–79	791	53	1,669	117	High
Trabert et al. (2019)🕈 [Table-fn TFN2]	2001–2003	Poland	Serology	55.5	55.6	244	58	556	103	High
Trabert et al. (2019)🕈 [Table-fn TFN2]	2001–2003	Poland	Serology	63.3	63.1	160	13	159	19	High
Skarga et al. (2023)🕈 [Table-fn TFN2]	1983–2016	Finland	Serology	15–45	16–45	484	140	484	127	High

✞ These studies had a case–control design;

🕈 These studies had a nested case–control design.

NR: not reported; NOS: Newcastle-Ottawa Scale; PCR: polymerase chain reaction.

### Results of meta-analysis

**Figure 1:**
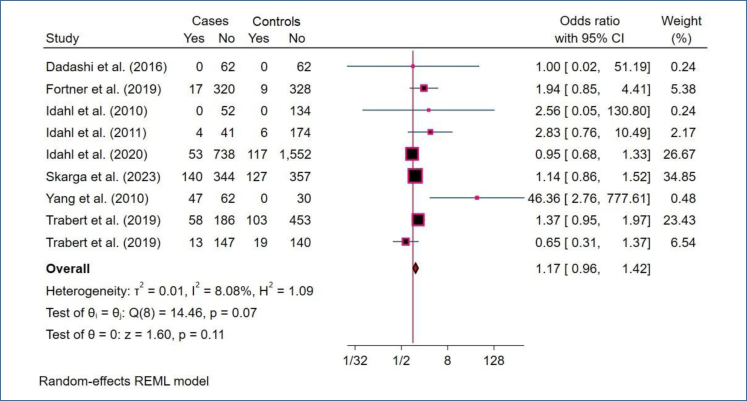
Forest plot presenting the pooled random-effects analysis for the association between *Mycoplasma genitalium i*nfection and ovarian cancer, displaying odds ratios with corresponding 95%CI.

**Figure 2:**
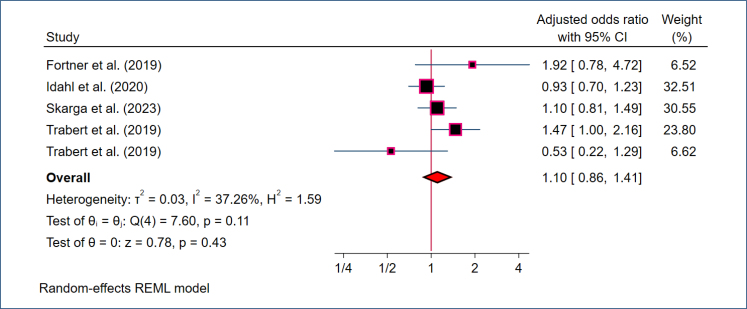
Forest plot displaying the pooled random-effects analysis of the association between *Mycoplasma genitalium* infection and ovarian cancer, with adjusted odds ratios and 95%CI for datasets using healthy controls.

The REM revealed no statistically significant association between MGI and ovarian cancer risk (OR 1.17, 95%CI 0.96–1.42; 95% PI 0.89–1.54; Figure 1). The PI suggests that future studies could plausibly observe a true effect ranging from an 11% decrease to a 54% increase in risk, reflecting between-study variation. Sensitivity analysis excluding the study by Idahl et al.^16^ showed a statistically significant association (OR 1.25, 95%CI 1.02–1.54; 95% PI 0.94–1.66; Supplementary Figure 2). This indicates a potential association, although the PI still encompasses the null value, highlighting residual uncertainty. Between-study heterogeneity was low (I^2^=8.08%, p=0.11), suggesting relatively consistent effect estimates across studies. A total of five studies estimated adjusted ORs; a REM on these adjusted ORs also indicated a non-statistically significant association between MGI and ovarian cancer risk (OR 1.10, 95%CI 0.86–1.41; I^2^=37.26%; Figure 2).

Subgroup analyses based on control group type showed non-significant associations in all subgroups (healthy controls: OR 1.15, 95%CI 0.96–1.38; borderline tumors: OR 0.56, 95%CI 0.20–1.61; and benign gynecological conditions: OR 2.76, 95%CI 0.37–20.54; Supplementary Figures 3, 4 and 5). The cumulative meta-analysis demonstrated an attenuating trend in the association as more recent studies were incorporated (Supplementary Figure 6). Assessment of publication bias using funnel plots and Egger's test showed a symmetrical funnel plot and no statistically significant evidence of bias (Egger's test: β=1.04, p=0.07; Supplementary Figure 7).

## DISCUSSION

This systematic review and meta-analysis explored the potential association between MGI and ovarian cancer risk. Our overall findings indicate that MGI is not significantly associated with ovarian cancer (OR 1.17, 95%CI 0.96–1.42). However, the results of the sensitivity analysis, which excluded one study^16^, revealed a statistically significant association (OR 1.25, 95%CI 1.02–1.54), suggesting that specific study characteristics or potential biases could have influenced the overall findings. The cumulative analysis also demonstrated a weakening trend in the association over time, implying that earlier studies reported stronger links between MGI and ovarian cancer compared to more recent ones. No significant publication bias was detected, supporting the robustness of our findings.

Our findings are largely consistent with those of a previous meta-analysis^6^; however, some key differences exist. While both studies reported non-significant associations overall, our study revealed a stronger and more consistent association between MGI and increased ovarian cancer susceptibility. This difference may be attributed to several factors. Our study incorporated sensitivity analysis, cumulative analysis, REM analysis on adjusted ORs, and subgroup analyses based on control type, while these analyses were absent in the previous meta-analysis. Furthermore, we excluded two studies with inadequate control groups^17,18^, which were included in the previous meta-analysis. This exclusion likely contributed to the observed discrepancies in findings.

While the exact molecular mechanisms remain under investigation, evidence supports the hypothesis that MGI contributes to a pro-tumorigenic environment via chronic inflammation, immune evasion, and tissue damage^12,19^. Chronic MGI is known to cause PID, which can extend to the fallopian tubes and ovaries, creating a persistent inflammatory response^11,20–22^. This chronic inflammation can lead to the production of reactive oxygen species (ROS) and pro-inflammatory cytokines such as interleukin-6 (IL-6) and tumor necrosis factor-alpha (TNF-α), both of which have been linked to DNA damage, impaired apoptosis, and increased cellular proliferation, key factors in oncogenesis^23,24^. Furthermore, MG possesses mechanisms to evade host immune responses, including the expression of variable surface proteins that allow it to escape immune detection and prolong infection^25,26^. This immune evasion can contribute to immunosuppression, reducing the effectiveness of immune surveillance and permitting the survival and growth of abnormal cells, which could eventually lead to malignant transformation^11^. Repeated cycles of epithelial injury and repair, driven by chronic infections like MG, can further exacerbate the risk of neoplastic transformation by weakening the epithelial barrier and allowing for direct invasion of pathogens or carcinogens into the ovarian tissue^11,27^. Together, these processes—chronic inflammation, immune evasion, and tissue damage—create an environment that may facilitate ovarian cancer development in individuals with persistent MGI^11,19,27^. Although more research is needed to fully elucidate these mechanisms, the existing evidence underscores the importance of chronic infections as potential risk factors for ovarian cancer.

Some strengths of this meta-analysis are the comprehensive search strategy in five global databases, the rigorous statistical methodology, no limitation for language or date, low heterogeneity, and no significant evidence of publication bias. Additionally, the sensitivity and cumulative analyses enhanced the reliability of the findings by exploring the stability of the results over time. However, several limitations should be considered. While heterogeneity and publication bias were low, the variability in diagnostic methods across studies remains a concern. Different diagnostic techniques, such as PCR versus serological assays, might have influenced the detection of MGI. PCR is generally more sensitive for detecting active infections, whereas serological methods may reflect past infections, potentially leading to inconsistencies in the pooled estimates. Although meta-regression or subgroup analyses by patient age, gender distribution, or tumor characteristics could elucidate sources of heterogeneity, such analyses were precluded by inconsistent reporting across studies. Future prospective studies should prioritize collecting and reporting stratified data (e.g., by cancer stage, treatment, or molecular subtypes) to enable more nuanced risk assessments. Another limitation is the small number of studies available for inclusion, which limited the statistical power to detect a significant overall association. Furthermore, despite adjustments for confounders in individual studies, residual confounding due to unmeasured factors, such as co-infections with other STIs or variations in sexual behavior, could not be fully accounted for. The sensitivity analysis indicated a significant association when one study was excluded, raising concerns about the influence of individual studies on the overall findings and the generalizability of the results. Lastly, most included studies were retrospective in design, which increases susceptibility to recall bias, potentially affecting the accuracy of reported associations.

## CONCLUSION

While our overall REM results do not provide conclusive evidence of a significant association between MGI and ovarian cancer, the findings from sensitivity analyses suggest a potential link that requires further exploration. Addressing these research gaps will be essential for developing a more comprehensive understanding of how STIs contribute to ovarian cancer development. Additionally, our findings highlight the need for larger, well-designed prospective studies to clarify the role of MGI in ovarian cancer risk. Future research should focus on standardizing diagnostic criteria for MGI and examining the biological mechanisms by which chronic infections might contribute to ovarian carcinogenesis.

## Data Availability

The datasets generated and/or analyzed during the current study are available from the corresponding author upon reasonable request.
